# Transcranial Direct Current Stimulation for Parkinson's Disease: A Systematic Review and Meta-Analysis

**DOI:** 10.3389/fnagi.2021.746797

**Published:** 2021-10-28

**Authors:** Xiang Liu, Huiyu Liu, Zicai Liu, Jinzhu Rao, Jing Wang, Pu Wang, Xiaoqian Gong, Youliang Wen

**Affiliations:** ^1^Department of Rehabilitation Medicine, Yuebei People's Hospital, Shaoguan, China; ^2^School of Rehabilitation Medicine, Gannan Medical University, Ganzhou, China; ^3^Department of Rehabilitation Medicine, The Seventh Affiliated Hospital, Sun Yat-sen University, Shenzhen, China; ^4^Yuebei People's Hospital, Shaoguan, China

**Keywords:** transcranial direct current stimulation, Parkinson's disease, meta-analysis, review, motor function, cognitive function

## Abstract

Background: Parkinson's disease is a common neurodegenerative disorder with motor and non-motor symptoms. Recently, as adjuvant therapy, transcranial direct current stimulation (tDCS) has been shown to improve the motor and non-motor function of patients with Parkinson's disease (PD). This systematic review aimed to evaluate the existing evidence for the efficacy of tDCS for PD. We included English databases (PubMed, the Cochrane Library, Embase, and Web of Science) and Chinese databases [Wanfang database, China National Knowledge Infrastructure (CNKI), China Science and Technology Journal Database (VIP), and China Biology Medicine (CBM)] without restricting the year of publication. Twenty-one tDCS studies, with a total of 736 participants, were included in the analysis. Two independent researchers extracted the data and characteristics of each study. There was a significant pooled effect size (−1.29; 95% CI = −1.60, −0.98; *p* < 0.00001; *I*^2^ = 0%) in the Unified PD Rating Scale (UPDRS) I and the Montreal cognitive assessment (SMD = 0.87, 95% CI = 0.50 to 1.24; *p* < 0.00001; *I*^2^ = 0%). The poor effect size was observed in the UPDRS III scores (SMD = −0.13; 95% CI = −0.64, 0.38; *p* = 0.61; *I*^2^ = 77%), and similar results were observed for the timed up and go (TUG) test, Berg balance scale, and gait assessment. The results of this meta-analysis showed that there was insufficient evidence that tDCS improves the motor function of patients with PD. However, tDCS seemed to improve their cognitive performance. Further multicenter research with a larger sample size is needed. In addition, future research should focus on determining the tDCS parameters that are most beneficial to the functional recovery of patients with PD.

## Introduction

Parkinson's disease is a common neurodegenerative disease in the elderly and its characteristic pathological changes are the progressive degeneration of the dopaminergic neurons in the substantia nigra and the significant decrease in the dopamine secretion of the striatum (Berg et al., 2014; Beretta et al., 2020a). Parkinson's disease (PD) is uncommon before 50 years of age in men and women, and the prevalence, morbidity, and death rates associated with this condition increase with age. In addition, men are more susceptible to develop this disease than women, and the incidence is 1.4 times higher than that in women. By the age of 60, the prevalence rate is ~0.5%, while the prevalence rises to ~3.9% in elderly individuals between 85 and 89 years of age (Dorsey et al., 2018). In China, the prevalence of PD among people over 65 years of age is ~1.7% (Zhang et al., 2005). The cardinal symptoms of PD include motor- and non-motor-related features. The motor symptoms include bradykinesia, rigidity, and static tremor, as well as postural and gait disorder (Jankovic, 2008). The common disabled non-motor symptoms in patients with PD mainly include emotional and cognitive disorders (Ransmayr, 2015). These symptoms can lead to dysfunctions such as balance disorders, cognitive impairment, and dysphagia, which reduce the ability for self-care in daily life and may even lead to death, increasing the economic burden on the family and society.

The treatment of dysfunctions in patients with PD requires comprehensive therapy and multidisciplinary participation, including movement therapy, dopamine replacement therapy, and the combined use of anticholinergic agents and deep brain stimulation (Goodwill et al., 2017). However, the effects of the medication may diminish over time (Jankovic and Stacy, 2007). These may include movement symptoms and fluctuations (Jankovic, 2008) and obsessive behaviors (Raja and Bentivoglio, 2012), as well as an increased risk of developing dementia (Gray et al., 2015). Adaptive deep brain stimulation has shown great potential in the treatment of PD, but its applicability in cognitive and other psychiatric disorders remains uncertain (Beudel and Brown, 2016; Guidetti et al., 2021). Therefore, alternative interventions should be explored.

In recent years, a growing number of researchers have paid more and more attention to the study of non-invasive brain stimulation techniques on the function of PD, such as transcranial direct current stimulation (tDCS). tDCS has two electrodes, an anode, and a cathode, which provide constant direct currents on the scalp. It has been shown to induce changes in the resting membrane potential of the cerebral cortex and change the excitability of neurons (Nonnekes et al., 2014). The anode increases the excitability of the cortical tissues, and the cathode decreases the excitability (Broeder et al., 2015; Lefaucheur et al., 2017), which may induce the release of neurotransmitters and increase the extracellular dopamine levels, as has been demonstrated in animal models (Tanaka et al., 2013). This may facilitate signal transduction in brain tissue. In addition, studies have shown that tDCS on the cognitive regions of the cerebral cortex could improve cortical excitability, and affect cognitive networks (Miniussi et al., 2013). tDCS has been suggested to improve cognitive ability (Boggio et al., 2006; Doruk et al., 2014; Biundo et al., 2015) and verbal fluency (Pereira et al., 2013), and cerebellar tDCS can activate specific neural networks and strengthen the regulation of behavioral responses associated with emotion-related stimuli (Ruggiero et al., 2021). However, studies have shown that between tDCS and sham intervention, there was no difference in the movement performance, reaction time, and self-assessment mobility in the Unified PD Rating Scale (UPDRS; Benninger et al., 2010).

Although tDCS shows great potential in treating PD, the results of the existing studies on the treatment of PD with tDCS were inconsistent, so it is necessary to systematically review the existing studies. This systematic review and meta-analysis aimed to summarize the available evidence to evaluate the clinical efficacy of tDCS in the treatment of PD.

## Methods

The systematic review and meta-analysis were conducted according to the preferred report items of systematic review and meta-analysis (Page et al., 2021).

### Eligibility Criteria

#### Study Types

Only relevant randomized controlled trials (RCTs) were included to investigate the efficacy of tDCS in PD treatment. Comments, case reports, quasi-RCTs, animal experiments, or non-RCTs were excluded.

#### Participants

According to diagnostic criteria (National Collaborating Centre for Chronic, 2006), participants diagnosed with PD were included in this review. There were no restrictions on age, gender, or race.

#### Intervention

The studies included tDCS intervention alone or in combination with any other interventions, including sham stimulation, Western medical treatment, or rehabilitation. Except for the tDCS intervention in the experimental trial, the interventions in the experimental and comparison trials are the same.

#### Outcomes

The primary outcomes were the motor and non-motor function assessments, including the UPDRS, timed up and go test (TUG), Berg balance scale (BBS), gait assessment, mini-mental state examination (MMSE), Montreal cognitive assessment (MoCA), PD-cognitive rating scale (PD-CRS), and PD Quality of Life Questionnaire-39 (PDQ-39).

### Search Strategy

The search was performed by restricting the language to English and Chinese without restricting the year of publication. Two of the authors (Xiang Liu and Huiyu Liu) created a search strategy, which was approved by all authors. To identify all potentially relevant studies, we searched the following databases: English databases: PubMed, the Cochrane Library, Embase, and Web of Science. Chinese databases: Wanfang database, China National Knowledge Infrastructure (CNKI), China Science and Technology Journal Database (VIP), and China Biology Medicine (CBM). All searches were conducted in June 2021 and covered the databases from their inception. The search terms were (a) “Parkinson's” or “Parkinson's disease” or “PD” or “idiopathic Parkinson's disease” or “IPD” or “primary Parkinson's syndrome”; (b) “transcranial direct current stimulation” or “tDCS”; and (c) “randomized controlled trials” or “RCTs” or “controlled clinical trial” or “randomized” or “randomly” or “trial.”

### Study Selection

The researchers (Xiang Liu and Huiyu Liu) scanned the title, abstract, and keywords of the articles found in the electronic search and excluded the irrelevant articles. Then, we obtained the full text of the included articles; subsequently, the full text of the potential studies was evaluated to determine their acceptability. All the differences and opinions were resolved through a discussion between the two researchers, and the research was selected according to the inclusion criteria. A third reviewer (Wang Pu or Youliang Wen) was consulted to resolve differences in data extraction.

### Data Extraction

We prepared a data extraction table that included the authors, publication date, and the characteristics (numbers, gender, age, and PD durations, and others), experimental group, control group, stimulation area, intensity, duration, frequency, and period of treatment, outcomes, and adverse events. It was defined that, in the normal use of qualified tDCS, any deleterious event occurring that results in human harm and is not related to the anticipated effects of tDCS.

### Assessment of the Risk of Bias in the Included Studies

The Cochrane bias risk assessment tool (Higgins et al., 2011) was used to assess the methodological quality. Two reviewers (Xiang Liu and Huiyu Liu) independently assessed the risk of bias of each included study according to the following characteristics: random sequence generation, allocation concealment, blinding, completeness of outcome data, selective outcome reporting, and other biases. The risks were classified into three levels: low risk of bias, unclear risk of bias, and high risk of bias. Any difference was resolved through discussions, and if consensus was not reached, the third reviewer (Pu Wang or Youliang Wen) was consulted.

### Statistical Analyses

The review Manage 5.3 software (Cochrane, London, United Kingdom) was used to conduct the statistical analyses of the overall and subgroup treatment effects of tDCS intervention in PD. For continuous data, the mean difference (MD) or standardized mean difference (SMD) was used for analysis. The dichotomous data analysis was calculated using the risk ratio (RR), and for both, 95% CIs was assessed using the *Z*-test. The heterogeneity between each group was tested using the *I*^2^ test and Cochran's *Q* statistic (Zintzaras and Ioannidis, 2005). Less than 25% of the *I*^2^ values indicate low heterogeneity, 25–50% indicate moderate heterogeneity, and >50% indicate high heterogeneity. A random-effects model was applied if high heterogeneity was observed (*p* < 0.05 or *I*^2^ > 50%); otherwise, a fixed-effects model was applied (*p* > 0.05 or *I*^2^ <50%). When significant heterogeneity was present, subgroup analysis and sensitivity analysis were performed on the data, and if the source of heterogeneity could not be determined, descriptive analysis was performed.

## Results

### Study Classification

**Figure 1:**
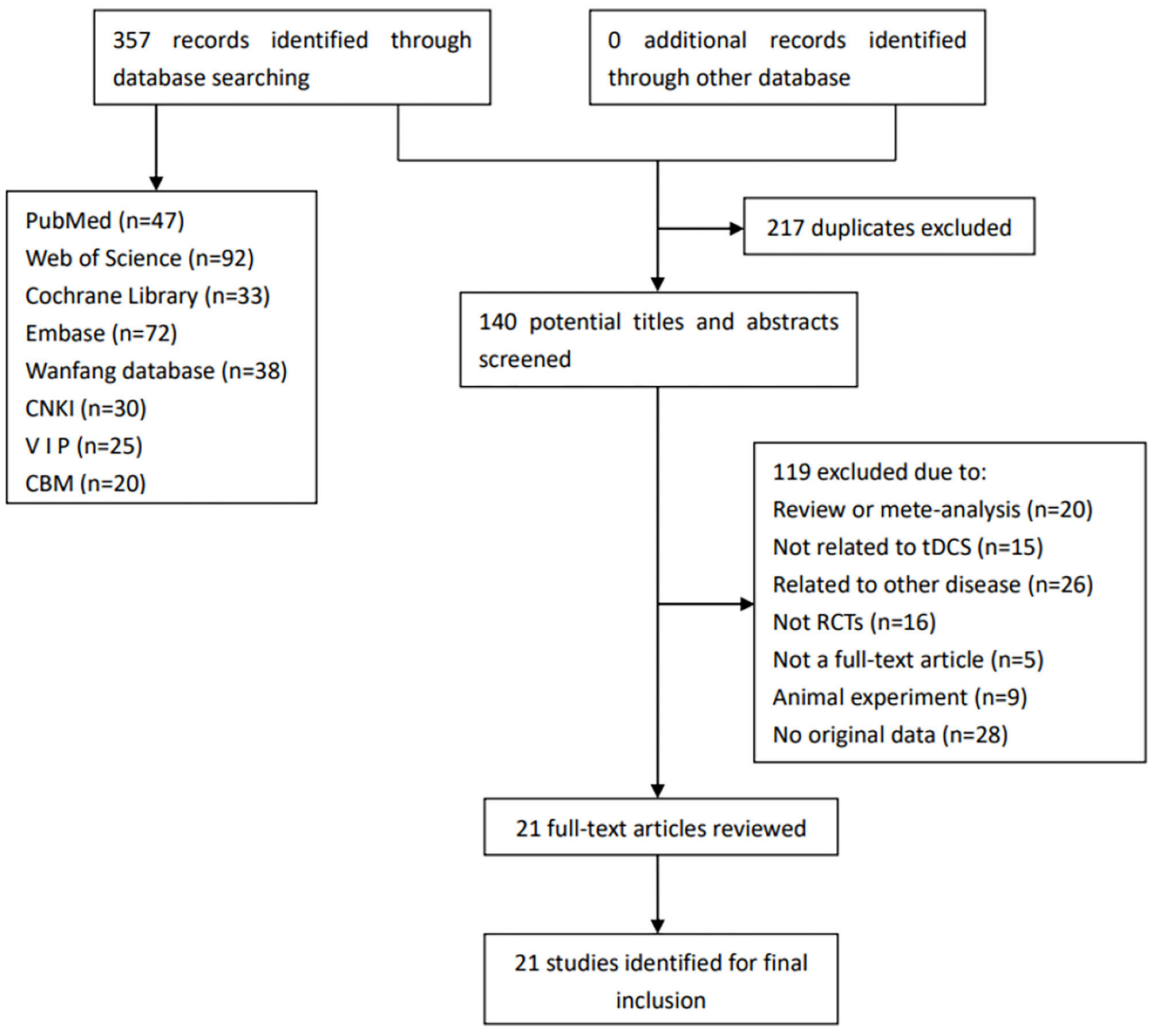
Flow diagram for the selection of the included studies. CBM, China Biology Medicine; CNKI, China National Knowledge Infrastructure; RCTs, randomized controlled trials; tDCS, transcranial direct current stimulation; VIP, China Science and Technology Journal Database.

Figure 1 shows the flow diagram for the selection of the included studies.

We searched out a total of 357 records from the relevant databases. First, 217 duplicated records were excluded, and 140 remained. Then, we read the articles carefully. In addition, 119 articles were excluded for the following reasons: review or meta-analysis (*n* = 20), not related to tDCS (*n* = 15), related to other disease (*n* = 26), not RCTs (*n* = 16), not a full-text article (*n* = 5), animal experiment (*n* = 9), and no original data (*n* = 28). Finally, 21 studies were eventually included in this review.

### Characteristics of the Included Studies

The participants and the methodological characteristics of the included studies are shown in Tables 1, 2. A total of 21 articles (all in all, 736 patients with PD) were included in this systematic review, of which 66.7% were by foreign authors and the remaining 33.3% were reported in a Chinese database. Twelve out of the 21 studies reported the drug status of participants, while the remaining nine did not mention the medication status. Among the 21 included studies, all received active tDCS or sham tDCS alone, or in combination with other treatments. The stimulation area of the brain includes the dorsolateral prefrontal cortex (DLPFC), the primary motor cortex (M1), prefrontal cortex (PFC), premotor cortex (PMC), and central zero (Cz) position. Among these areas, the DLPFC was the most frequently stimulated site, with 13 studies out of 21, followed by M1 (four studies), PFC, PMC, and Cz position. Finally, 17 studies applied repeated sessions of tDCS protocols, three studies used a single session of tDCS, and one study did not report.

**Table 1:** Characteristics of participants included in the studies.

**References**	**Total *N***	**Gender (M/F)**	**Age (year)**	**PD duration**	**UPDRS III**	**LED (mg/d)**
Wu and Wu (2016)	100	G1:27/23 G2:26/24	G1:60.4 ± 2.4 G2:59.7 ± 2	NR	G1:23.9 ± 6.5 G2:25.1 ± 7.1	NR
Criminger et al. (2018)	16	12/4	68.13 ± 9.76	8.69 ± 9.76	23.44 ± 9.73	NR
Qiao et al. (2019)	49	G1:11/14 G2:15/9	G1:63.00 ± 9.20 G2:62.04 ± 9.69	G1:5.22 ± 4.46 G2:6.07 ± 5.25	G1:20.32 ± 5.57 G2:22.08 ± 7.51	G1:340.64 ± 16.78 G2:351.63 ± 128.62
Manenti et al. (2016)	20	G1:4/6 G2:7/3	G1:69.0 ± 6.1 G2:69.1 ± 5.6	G1:7.1 ± 3.6 G2:7.8 ± 4.2	G1:27.8 ± 13.9 G2:27.6 ± 18.9	G1:524.6 ± 179.1 G2:815.7 ± 590.9
Qiao and Yan (2018)	30	G1:7/8 G2:9/6	G1:62.00 ± 10.26 G2:58.40 ± 9.13	G1:4.03 ± 4.94 G2:3.51 ± 2.51	G1:18.33 ± 4.30 G2:19.47 ± 5.79	G1:255.20 ± 79.13 G2:275.13 ± 77.47
Li et al. (2018)	56	G1:15/13 G2:14/14	G1:64.32 ± 5.59 G2:64.39 ± 5.50	G1:1.19 ± 0.57 G2:1.28 ± 0.56	G1:34.5 ± 13.77 G2:34.6 ± 12.85	NR
Zhu (2020)	70	G1:24/11 G2:23/12	G1:77.06 ± 3.23 G2:77.11 ± 0.25	G1:3.62 ± 2.15 G2:3.95 ± 2.17	NR	NR
Yang and He (2017)	94	G1:26/21 G2:27/20	G1:62.4 ± 4.1 G2:62.8 ± 4.3	NR	G1:23.56 ± 6.39 G2:24.62 ± 7.62	NR
Wu and Li (2020)	54	G1:16/12 G2:14/12	G1:61.0 ± 11.6 G2:62.6 ± 12.2	G1:5.8 ± 2.6 G2:5.7 ± 3.5	NR	G1:424.9 ± 96.9 G2:420.4 ± 90.7
Manenti et al. (2014)	10	6/4	67.1 ± 7.2	8.1 ± 3.5	133.3 ± 5.7	749.2 ± 445.5
Schabrun et al. (2016)	16	G1:8/0 G2:2/6	G1:72 ± 4.9 G2:63 ± 11.0	G1:6.9 ± 4.4 G2:4.6 ± 3.9	G1:47.7 ± 7.5 G2:37.7 ± 9.8	G1:730 ± 341 G2:523 ± 398
Fernandez-Lago et al. (2017)	18	11/7	56.67 ± 11.63	6.17 ± 3.65	NR	733.2 ± 496.2
Lattari et al. (2017)	17	13/4	69.18 ± 9.8	7.06 ± 2.70	18.0 ± 8.96	748.29 ± 343.80
Yotnuengnit et al. (2018)	53	G1:11/6 G2:12/6 G3:10/8	G1:68.2 ± 9.8 G2:62.7 ± 8.8 G3:64.4 ± 7.8	G1:9.4 ± 5.3 G2:6.6 ± 3.6 G3:7.9 ± 3.9	G1:11.94 ± 4.68 G2:11.17 ± 3.97 G3:10.89 ± 4.75	G1:829.0 ± 360.6 G2:912.0 ± 472.9 G3:849.1 ± 397.1
Costa-Ribeiro et al. (2017)	22	G1:8/3 G2:7/4	G1:61.1 ± 9.1 G2:62.0 ± 16.7	G1:6.1 ± 3.8 G2:6.3 ± 3.7	G1:19.0 ± 4.9 G2:17.6 ± 5.1	G1:740.9 ± 924.3 G2:890.9 ± 836.0
Swank et al. (2016)	10	8/2	68.7 ± 10.2	7.9 ± 7.1	NR	NR
Kaski et al. (2014b)	16	NR	NR	NR	NR	NR
Lawrence et al. (2018)	28	G1:2/5 G2:4/3 G3:3/4 G4:2/5	G1:63.57 ± 15.68 G2:68.14 ± 8.69 G3:72.29 ± 6.21 G4:72 ± 6.45	G2:6.79 ± 4.6 G2:5.29 ± 4.23 G3:5.36 ± 4.14 G4:5.50 ± 5.66	NR	G1:350.71 ± 322.37 G2:295 ± 313.40 G3:292.88 ± 274.51 G4:573.29 ± 586.25
Benninger et al. (2010)	15	G1:9/4 G2:7/5	G1:53.56 ± 11.5 G2:55.1 ± 8.7	G1:10.6 ± 7.1 G2:9.1 ± 3.3	NR	NR
Bueno et al. (2019)	20	8/12	64.45 ± 8.98	7.80 ± 5.32	22.35 ± 6.77	NR
Manenti et al. (2018)	22	G1:5/6 G2:7/4	G1:65.5 ± 6.4 G2:63.8 ± 7.1	G1:6.2 ± 3.9 G2:7.6 ± 3.4	G1: 26 ± 10.3 G2: 22.7 ± 7.8	G1:618.6 ± 304.4 G2:559.8 ± 306.5

*M, Male; F, Female; PD, Parkinson's disease; UPDRS, Unified Parkinson's disease rating scale; UPDRS III, motor part of Unified Parkinson's disease rating scale; LED, Levodopa equivalent dose; G, group; G1, Experimental group 1; G2, Control group 1, G3, Control group2, G4, Experimental group 2, NR, Not Reported*.

**Table 2:** tDCS protocols of the included studies.

**References**	**Treatment**	**Stimulation area**	**Stimulation parameters (intensity/duration/size/session)**	**Outcome**	**Follow-up**	**Adverse events**
Wu and Wu (2016)	G1: tDCS+CM G2:CM	A: L-DLPFC C: CSA	1 mA,10 min, NR, 30	UPDRS, UPDRS I, UPDRS II, UPDRS III	NR	NR
Criminger et al. (2018)	G1: A-tDCS G2: sham-tDCS	A: L-DLPFC C: R-DLPFC	2 mA, 20 min, 15 cm^2^, 4	TUG	NR	Headache
Qiao et al. (2019)	G1: A-tDCS+CM+PT G2: sham-tDCS+CM+PT	A: L-DLPFC C: CSA	2 mA, 20 min, 35 cm^2^, 5	BBS	NR	NR
Manenti et al. (2016)	G1: A-tDCS+PT G2: sham-tDCS+ PT	A: L or R-DLPFC C: CSA	2 mA, 25 min, 35 cm^2^, 10	UPDRS III, TUG, PDQ-39, PD-CRS	3 m	NR
Qiao and Yan (2018)	G1: A-tDCS+CM+PT G2: sham-tDCS+CM+PT	A: L-DLPFC(F3) C: CSA(Fp2)	2 mA, 20 min, NR, 5	TUG, Velocity, Cadence, Stride width	NR	NR
Li et al. (2018)	G1: A- tDCS G2: sham-tDCS	A: Cz position C: Midpoint of the line at the superior orbital margin	2 mA, 20 min, 35 cm^2^, 56	MoCA	NR	NR
Zhu (2020)	G1: tDCS+ CM G2: CM	A: L-DLPFC C: CSA	2 mA, 20 min, NR, 20	MoCA, MMSE	NR	NR
Yang and He (2017)	G1: tDCS+ CM G2: CM	A: L-DLPFC C: CSA	1 mA, 20 min, NR, 30	UPDRS, UPDRS I, UPDRS II, UPDRSIII	NR	Insomnia Dizziness Constipation
Wu and Li (2020)	G1: tDCS+ CM G2: CM	A: Bilateral prefrontal lobe (Fz) and DLPFC (F3/F4) C: Bilateral shoulder	1.2 mA, 20 min, 24.75 cm^2^, 20	PDQ-39	NR	NR
Manenti et al. (2014)	G1: A- tDCS G2: sham-tDCS	A: L or R-DLPFC C: CSA	2 mA, 7 min, 35 cm^2^, 2	TUG	NR	Inferred that all of the subjects tolerated the stimulation well
Schabrun et al. (2016)	G1: A- tDCS +GT G2: sham-tDCS+GT	A: L-M1 C: CSA	2 mA, 60 min, 35 cm^2^, 9	Velocity, Cadence, Stride length	12 w	One participant experienced strong tingling over the site of one electrode and a momentary flash of light. The sensations lasted ~5 s. No other events or symptoms reported
Fernandez-Lago et al. (2017)	G1: A- tDCS +TT G2: sham- tDCS +TT	A: Motor cortex C: CSA	2 mA, 20 min, 3.5 cm^2^, 4	Velocity, Stride length, stride frequency	NR	NR
Lattari et al. (2017)	G1: A-tDCS G2: sham-tDCS	A: L-DLPFC (F3) C: CSA (FP2)	2 mA, 20 min, 35 cm^2^, 1	BBS, TUG	NR	There were not any drop-outs from the trial and all participants were included for analysis
Yotnuengnit et al. (2018)	G1: A-tDCS+PT G2: sham-tDCS+PT G3: A- tDCS	A: Cz position C: CSA	2 mA, 30 min, 35 cm^2^, 6	Velocity, Stride length, Stride width, Cadence, UPDRS II, UPDRS III	2 w, 4 w; 8 w	During the intervention period, two subjects, who received the anodal tDCS intervention for the first time, reported a burning sensation on their forehead where the electrode was attached
Costa-Ribeiro et al. (2017)	G1: A- tDCS+GT G2: sham-tDCS+GT	A: placed 2 cm anterior to the vertex (Cz position, EEG 10/20 system) C: AHAS	2 mA, 13 min, NR, 10	TUG, Velocity, Cadence Stride-length, UPDRS III, BBS, PDQ-39	1 m	No adverse events were reported by any of the participants
Swank et al. (2016)	G1: A- tDCS G2: sham-tDCS	A: L-DLPFC C: R-DLPFC	2 mA, 20 min, NR, 1	TUG	NR	All participants completed the study.
Kaski et al. (2014b)	G1: A- tDCS+GBT G2: sham-tDCS+GBT	A: M1 C: Inion	2 mA, 15 min, 40 cm^2^ (A) 16 cm^2^ (C) NR	Velocity, Stride length, TUG	NR	NR
Lawrence et al. (2018)	G1: tDCS+CT G2: CT G3: Nothing G4: tDCS	A: L-DLPFC C: Over the left eye	1.5 mA, 20 min, 35 cm^2^, 12	PDQ-39, PD-CRS, MMSE	12 w	NR
Benninger et al. (2010)	G1: A- tDCS G2: sham-tDCS	A: premotor and motor or PFC C: PFC or premotor and motor	2 mA, 20 min, 24.5 cm^2^ (A) 25 cm^2^ (C), 8	UPDRS, UPDRS III	1 m; 3 m	Small first degree burns over the mastoids partially covering the earlobes with reduced contact surface resulting in an increased current density which healed completely within 3 days
Bueno et al. (2019)	G1: A- tDCS G2: sham-tDCS	A: L-DLPFC (F3) C: right orbital frontal cortex (Fp2)	2 mA, 20 min, 35 cm^2^, 1	TUG, Velocity, Cadence	NR	All subjects demonstrated good tolerability toward the application of the stimulation without exhibiting any adverse effects
Manenti et al. (2018)	G1: A- tDCS +CCT G2: sham- tDCS +CCT	A: L-DLPFC (F3) C: over the right Supraorbital area	2 mA, 25 min, 35 cm^2^, 10	UPDRS III, PDQ-39, PD-CRS	3 m	Not mentioned in the article

*tDCS, transcranial Direct Current Stimulation; A- tDCS, Active tDCS; A, Anode; C, Cathode; w, week; m, month; CM, Conventional Medicine; CSA, Contralateral Supraorbital area; AHSA, Affected Hemisphere Contralateral Supraorbital area; L, Left; R, Right; DLPFC, Dorsolateral Prefrontal Cortex; UPDRS, Unified Parkinson's disease rating scale; GT, Gait training; GBT, Gait and Balance Training. TT, Treading Training; PT, Physical therapy; TUG, Time Up and Go Test; CT, Cognitive Training; CCT, Computerized Cognitive Training. BBS, Berg Balance Scale; Cz, Central zero; PFC, Prefrontal cortex; PMC, Premotor cortex; M1, Primary motor cortex. PDQ-39, Parkinson's Disease Quality of Life Questionnaire-39; PD-CRS, Parkinson Disease-Cognitive Rating Scale; MoCA, Montreal Cognitive Assessment; MMSE, Mini-mental State Examination; NR, Not Reported*.

### Methodological Quality

**Figure 2:**
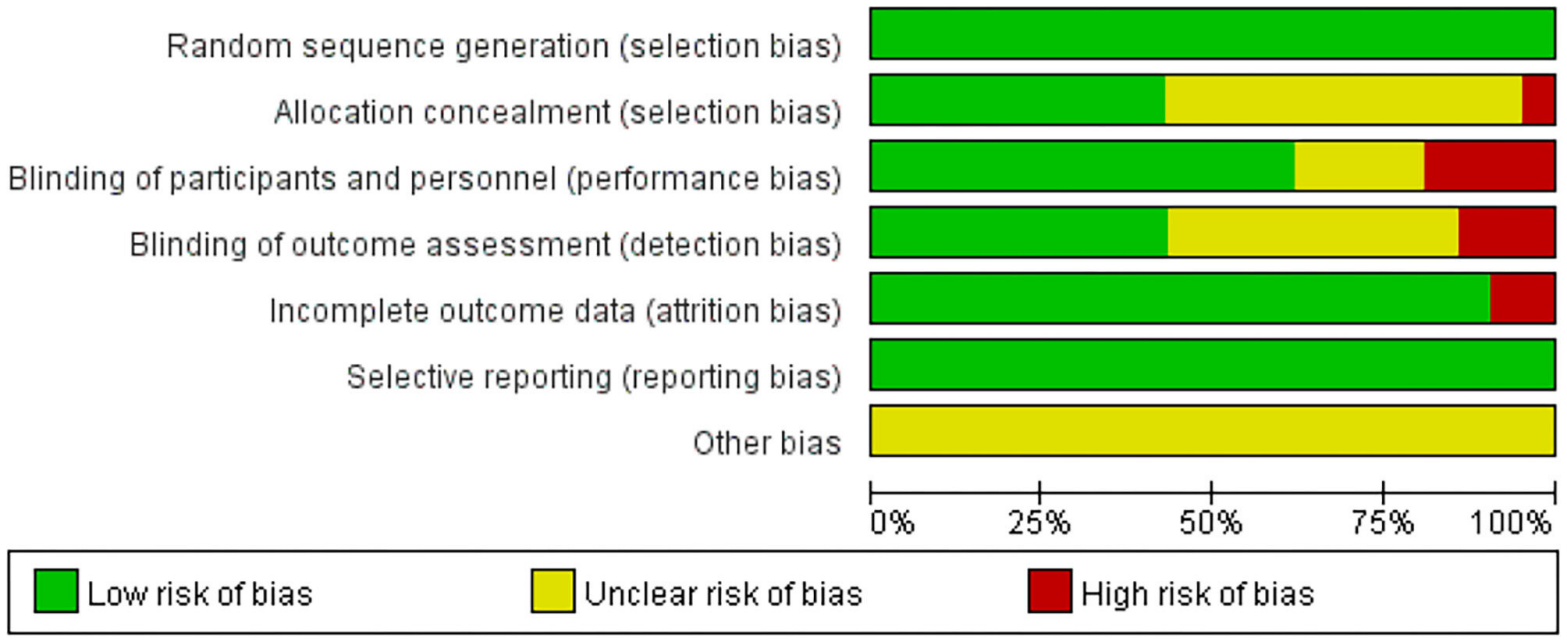
Risk of bias of the included studies.

**Figure 3:**
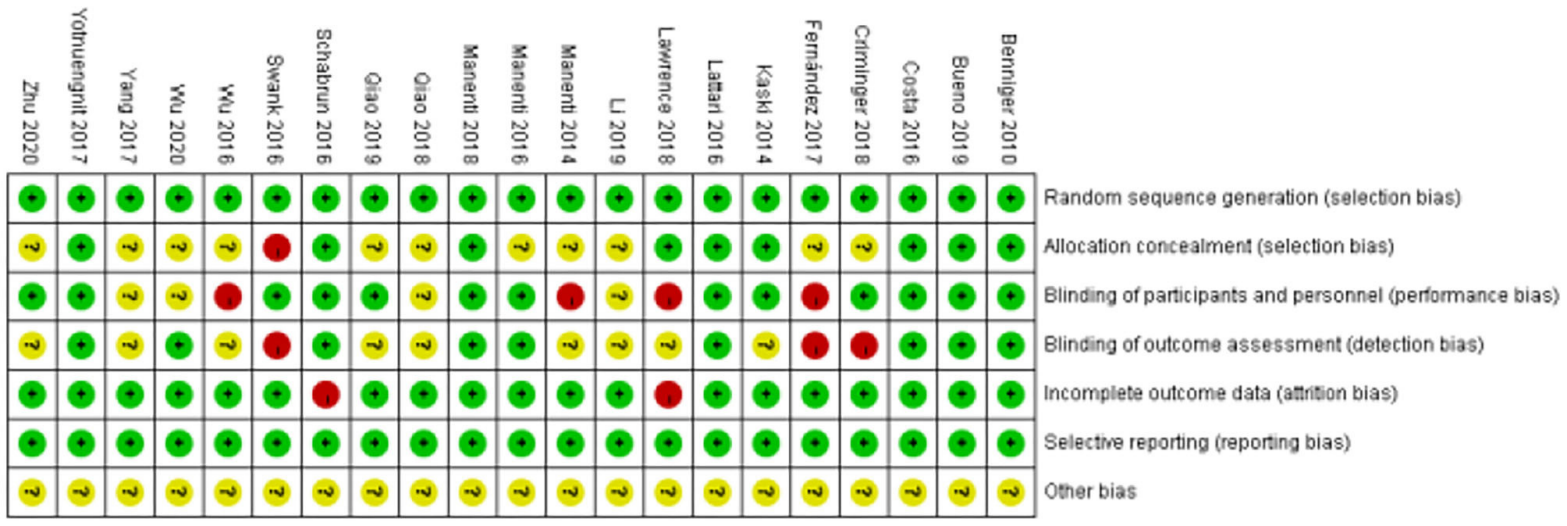
Risk of bias summary of the included studies.

The details of the risk of bias of all the included studies are shown in Figures 2, 3. All the articles used the random method to generate sequences. In the allocation concealment, only one study was high risk and nine studies were low risk. For the blinding of the outcome assessment, three retrieved studies were high risk and nine were unclear. During the evaluation process, we found that two articles mentioned patients who dropped out but were not included in the analysis, which may increase the risk of bias. In addition, all the studies clearly described selective reporting.

### Unified Parkinson's Disease Rating Scale III (UPDRS III)

**Figure 4:**
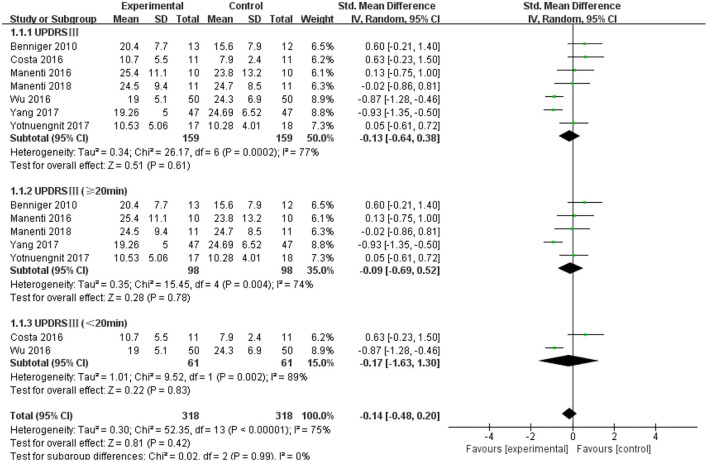
Effects of tDCS on the UPDRS III scores in patients with PD. Comparison between subgroups of tDCS duration. CI, confidence intervals; PD, Parkinson's disease; SD, standard deviation; tDCS, transcranial direct current stimulation; UPDRS, Unified Parkinson's Disease Rating Scale.

**Figure 5:**
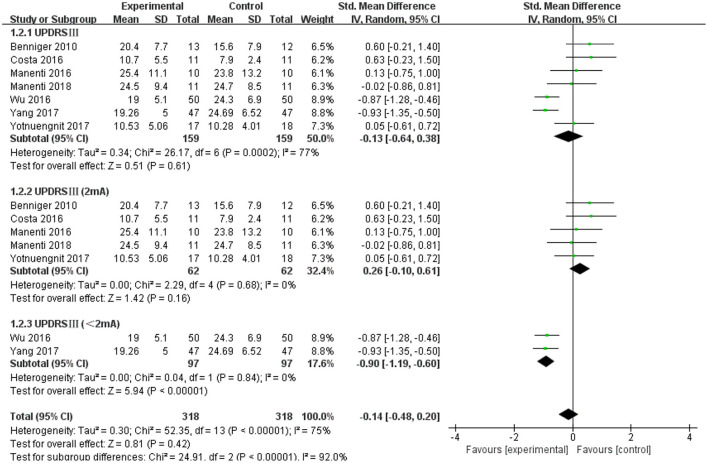
Forest plot of UPDRS III according to the subgroup of tDCS intensity. CI, confidence intervals; SD, standard deviation; tDCS, transcranial direct current stimulation; UPDRS, Unified Parkinson's Disease Rating Scale.

**Figure 6:**
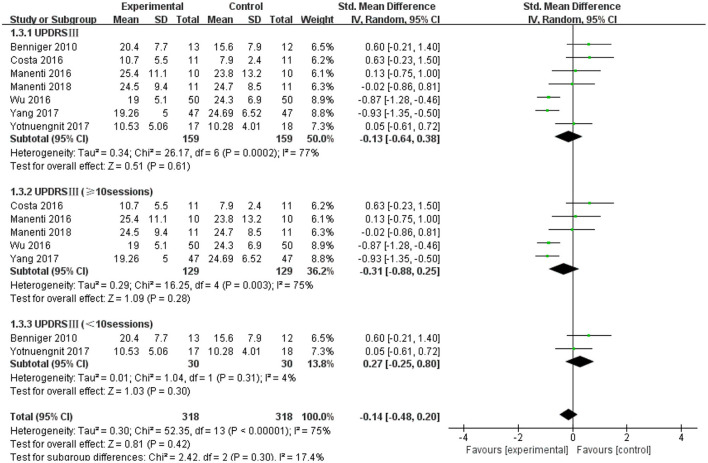
Forest plot of UPDRS III according to the subgroups of tDCS session. CI, confidence intervals; SD, standard deviation; tDCS, transcranial direct current stimulation; UPDRS, Unified Parkinson's Disease Rating Scale.

The UPDRS III was used as an outcome measure in seven of the 21 studies. The UPDRS III scores of the seven studies showed a non-significant effect size (−0.13; 95% CI = −0.64,0.38; *p* = 0.61; *I*^2^ = 77%) (Figure 4). Due to the high heterogeneity (*I*^2^ = 77%), we performed a subgroup analysis of the stimulus parameters. For the duration of the stimulus, there were five articles ≥20 min (SMD = −0.09; 95% CI = −0.69, 0.52; *p* = 0.78) and two articles <20 min (SMD = −0.17; 95% CI = −1.63, 1.30; *p* = 0.83), all of which did not show a significant pooled effect size (Figure 4). For stimulus intensity, there were five articles at 2 mA and there was a non-significant pooled effect size (SMD =0.26; 95% CI = −0.10, −0.61; *p* = 0.16; *I*^2^ = 0%). Two articles measuring <2 mA showed a significant pooled effect size (SMD = −0.90; 95% CI = −1.19, −0.60; *p* < 0.00001; *I*^2^ = 0%) (Figure 5). The two studies were identical in terms of the stimulus location (left DLPFC and contralateral supraorbital area) and result evaluation (UPDRS), with different intensities. Wu and Wu (2016) used 1 mA current for 10 min, while Yang and He (2017) used 1 mA for 20 min. For the stimulation session, there were two articles with fewer than 10 sessions (SMD = 0.27; 95% CI = −0.25, 0.80; *p* = 0.30; *I*^2^ = 4%) and five articles with 10 or more sessions (SMD = −0.31; 95% CI = −0.88, 0.25; *p* = 0.28), none of which showed effectiveness (Figure 6).

### Time Up and go Test (TUG) and Berg Balance Scale (BBS)

**Figure 7:**
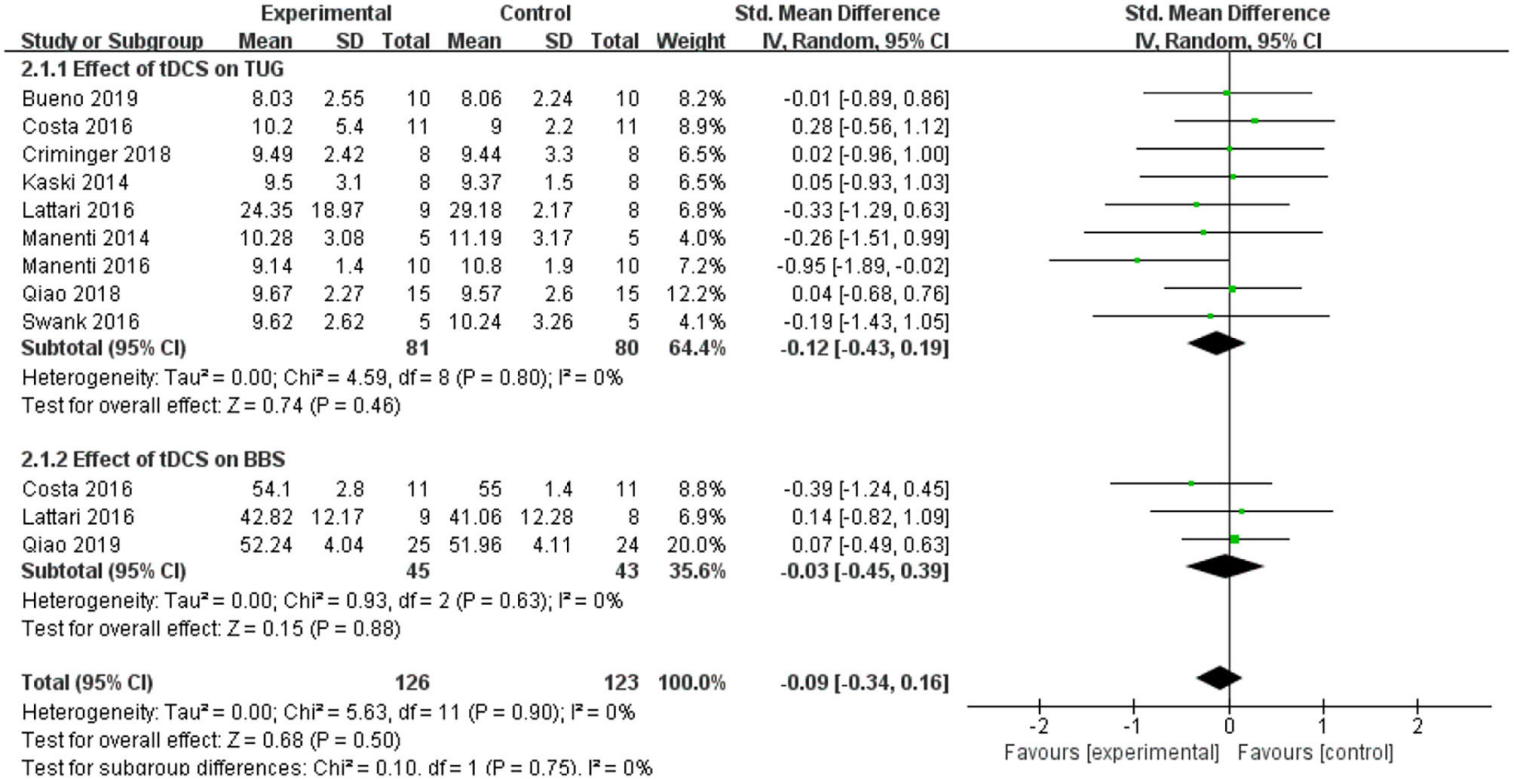
Forest plot of TUG and BBS. As shown in the Figure, tDCS did not improve the performance in the TUG and BBS significantly. BBS, Berg balance scale; CI, confidence intervals; SD, standard deviation; tDCS, transcranial direct current stimulation; TUG, timed up and go test.

The TUG test was used as an evaluation standard. Nine studies employed the TUG test. The meta-analysis showed an insignificant pooled effect size (−0.12; 95% CI = −0.43, 0.19; *p* = 0.46; *I*^2^ = 0%) (Figure 7). Three studies used the BBS. The result was non-significant as that of the TUG, and the pooled effect size was SMD = −0.03; 95% CI = −0.45 to 0.39; *p* = 0.88) (Figure 7).

### Velocity, Cadence, Stride Length, and Stride Width

**Figure 8:**
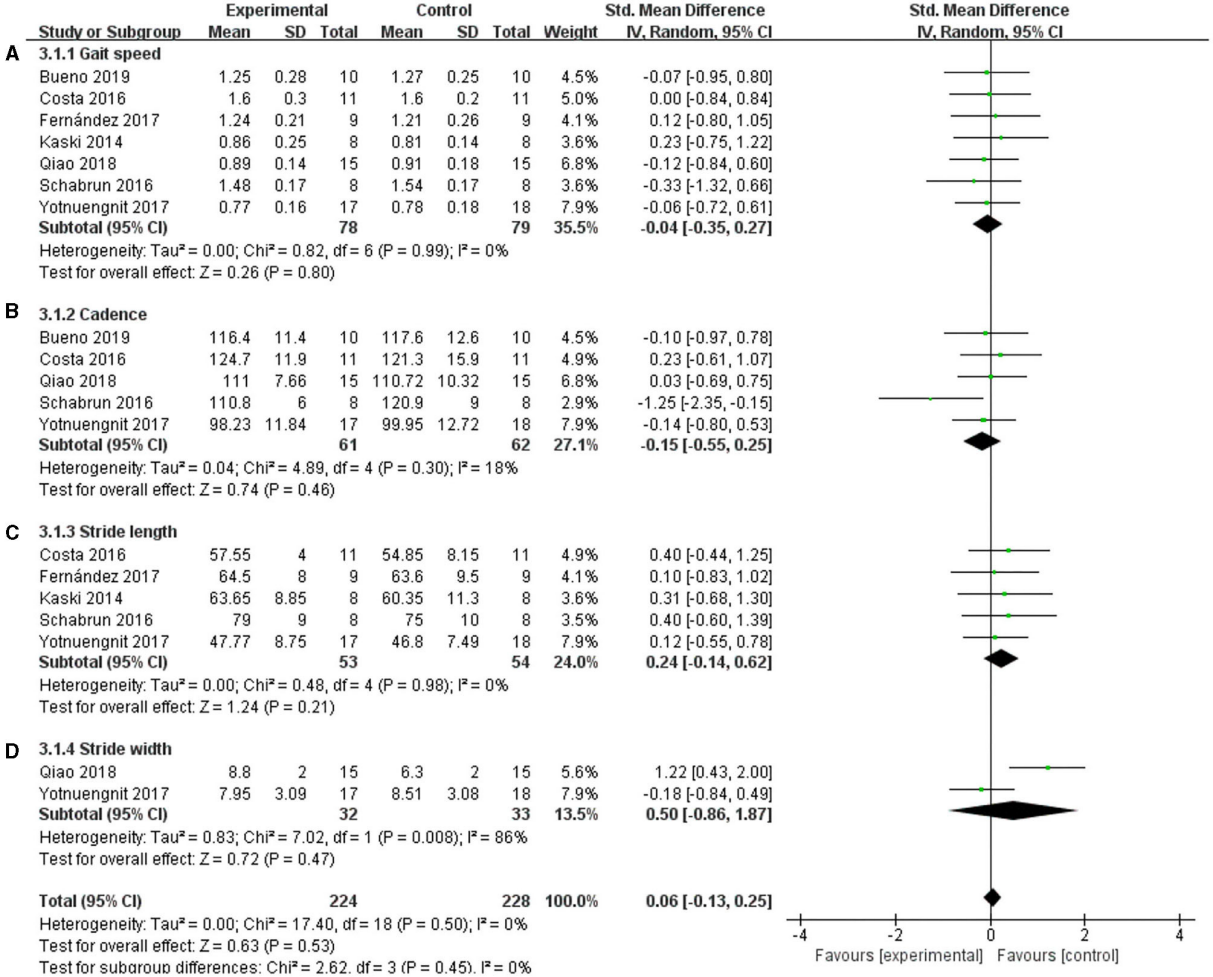
Effects of tDCS on gait scores in PD patients. **(A)** Effect of tDCS on gait speed in patients with PD. There was a non-significant pooled effect size (*p* = 0.80). **(B)** tDCS had a non-significant effect on the cadence of PD patients (*p* = 0.46). **(C)** tDCS had a non-significant effect on the stride length of PD patients (*p* = 0.21). **(D)** Effect of tDCS on the stride width of patients with PD. A non-significant pooled effect size was observed (*p* = 0.47). CI, confidence intervals; PD, Parkinson's disease; SD, standard deviation; tDCS, transcranial direct current stimulation; TUG, timed up and go test.

Gait parameters, including velocity, cadence, stride length, and stride width, were measured as the outcomes of tDCS in patients with PD. Seven studies used velocity, and a non-significant effect was shown (Figure 8A) (SMD = −0.04; 95% CI = −0.35, 0.27; *p* = 0.80; *I*^2^ = 0%). Five studies used cadence, and this meta-analysis showed a non-significant pooled effect size (−0.15; 95% CI = −0.55, 0.25; *p* = 0.46; *I*^2^ = 18%) (Figure 8B). Five studies reported stride length, and the results showed that tDCS was not effective in improving the step length of patients with PD. The effect size of SMD = 0.24, 95% CI was −0.14 to 0.62, *p* = 0.21, *I*^2^ = 0% (Figure 8C). Two studies used stride width as the outcome measure, and the meta-analysis of these data also showed no significant effect (SMD =0.50, 95% CI = −0.86 to 1.87; *p* = 0.47; *I*^2^ = 86%) (Figure 8D).

### MMSE, MoCA, PD-CRS, and UPDRS I

**Figure 9:**
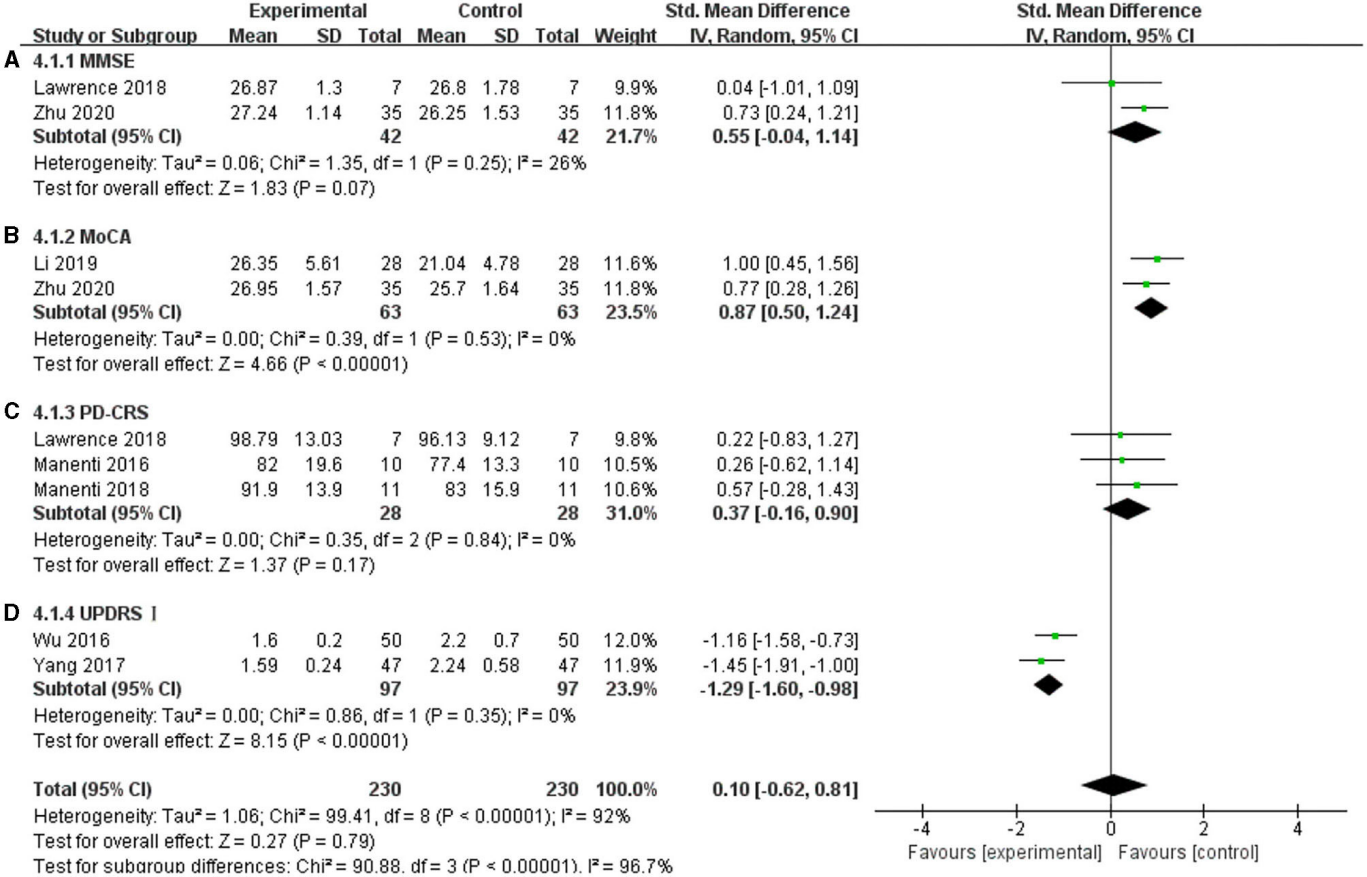
Effects of tDCS on cognition and emotion in patients with PD. **(A)** For the MMSE, there was a non-significant pooled effect size (*p* = 0.07). **(B)** For MoCA, two studies showed a significant pooled effect size (*p* < 0.00001). **(C)** tDCS had a non-significant effect on PD-CRS in PD patients (*p* = 0.17). **(D)** For UPDRS I, two studies showed a significant pooled effect size (*p* < 0.00001). CI, confidence intervals; MMSE, Mini-Mental State Examination; PD, Parkinson's disease; PD-CRS, Parkinson's disease-cognitive rating scale; SD, standard deviation; UPDRS, Unified Parkinson's disease rating scale.

The non-motor symptoms of PD mainly include cognitive dysfunction and emotional disorders, and they seriously affect the quality of the daily lives of patients with PD. In this meta-analysis, MoCA, MMSE, PD-CRS, and UPDRS I were used. The MoCA scale showed a significant effect on improving cognitive functions in the included articles, and the effect size was SMD = 0.87, 95% CI =0.50 to 1.24; *p* < 0.00001; *I*^2^ = 0% (Figure 9B). For UPDRS I, two studies showed a significant pooled effect size (−1.29; 95% CI = −1.60, −0.98; *p* < 0.00001; *I*^2^ = 0%) (Figure 9D). However, the MMSE and PD-CRS showed a non-significant pooled effect size. The MMSE was SMD = 0.55, 95% CI = −0.04 to 1.14; *p* = −0.07; *I*^2^ = 26% (Figure 9A), and the PD-CRS was SMD = 0.37; 95% CI = −0.16 to 0.90; *p* = 0.17; *I*^2^ = 0% (Figure 9C). These results may be due to the tDCS parameters and severity of the disease. Lawrence et al. (2018) used a 1.5 mA current for 20 min in 12 sessions; Zhu (2020) used 2 mA for 20 min in 20 sessions, while Manenti et al. (2016, 2018) used 2 mA for 25 min, for a total of 10 sessions. The duration of PD included in their studies varied greatly. The average duration was 3.785 ± 2.16 in Zhu's study (2020), 7.45 ± 3.9 in Manenti et al. (2016), 6.9 ± 3.65 in Manenti et al. (2018), and 5.81 ± 4.32 in Lawrence et al. (2018). Due to these differences, the effects of tDCS on these factors were inconsistent.

### PDQ-39 and UPDRS II

**Figure 10:**
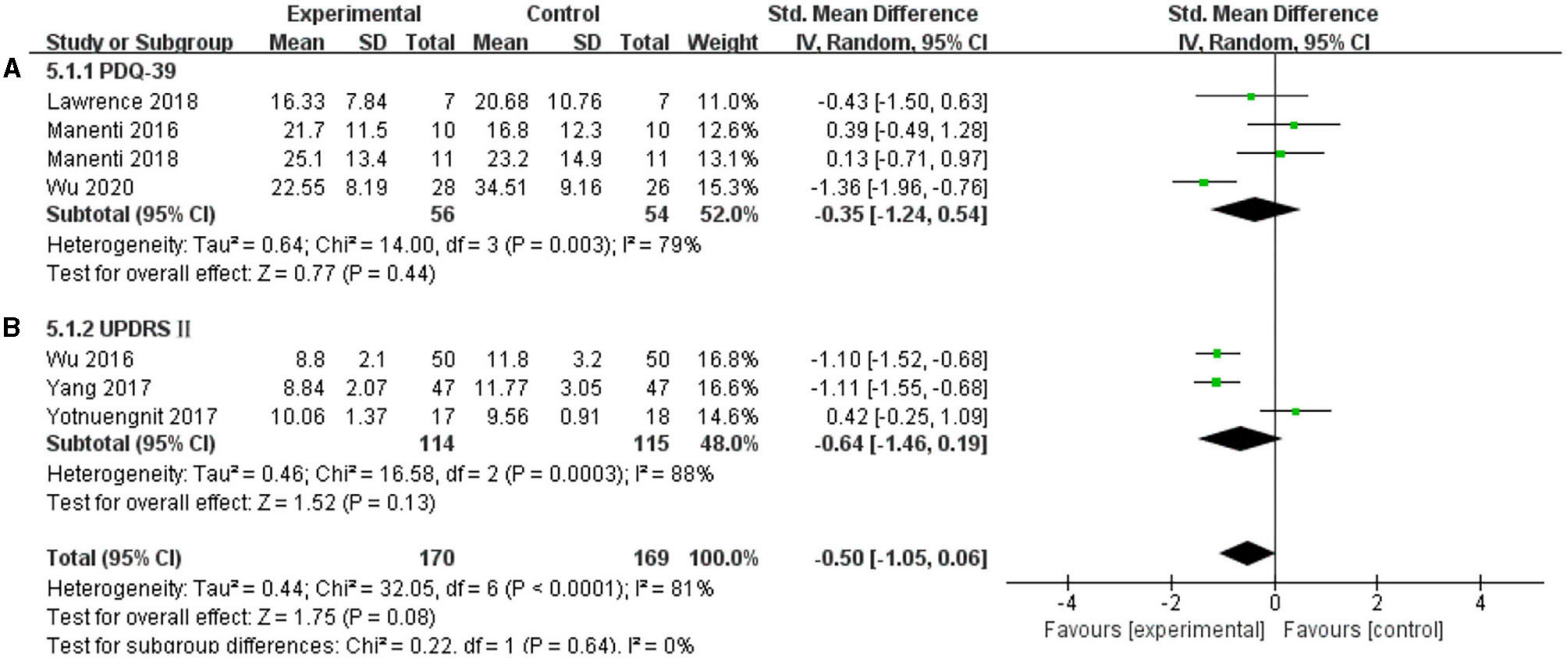
Effects of tDCS on the daily living scores of patients with PD. As shown in **(A,B)**, tDCS did not significantly improve the ability of daily living of patients with PD. CI, confidence intervals; PD, Parkinson's disease; PDQ-39, Parkinson's Disease Quality of Life Questionnaire-39; SD, standard deviation; UPDRS, Unified Parkinson's Disease Rating Scale.

In this systematic review, four studies were included to analyze whether tDCS can improve the self-care ability of patients with PD in daily life, and the results were non-effective (SMD = −0.35, 95% CI = −1.24 to 0.54; *p* = 0.44; *I*^2^ = 79%) (Figure 10A). Three studies selected UPDRS II as an outcome measure, and this meta-analysis found a non-significant pooled effect size (−0.64; 95% CI = −1.46, 0.19; *p* = 0.13; *I*^2^ = 88%) (Figure 10B).

### Adverse Events

Adverse events were reported in five studies. Yang and He (2017) reported that both the experimental group and the control group experienced adverse events, including insomnia, dizziness, postural hypotension, and constipation, with an incidence rate of 20.21%. In Criminger et al. (2018), one patient developed a headache. In Schabrun et al. (2016), one participant experienced a strong tingling and a momentary flash of light over the area of one electrode, and the sensations lasted for about 5 s. Yotnuengnit et al. (2018) mentioned that during the intervention period, two participants experienced a burning sensation on their forehead skin. In Benninger et al. (2010), one subject had a small number of first-degree burns. However, none of these adverse events resulted in serious consequences in any of the included studies.

## Discussion

The present systematic review of clinical studies of tDCS in PD aimed to evaluate the efficacy of tDCS as a clinical therapy for PD based on the existing evidence. In this review, the high heterogeneity of stimulation parameters does not allow us to firmly conclude that tDCS improves cognitive performance. In addition, it is uncertain whether tDCS can improve motor function in patients with PD. Further studies with larger sample sizes are needed to explore this possibility more thoroughly. Below, we discuss the effect of tDCS on motor and non-motor functions.

The 21 studies included were consistent in terms of the tDCS areas. In all studies, electrodes were placed to target the brain regions associated with motor or cognitive function. The studies aimed at improving cognition in the DLPFC or PFC, and the studies aimed to improve motor function in patients with PD employed electrodes at the M1 or premotor cortex. Fourteen studies used the DLPFC as the stimulation area. The DLPFC is considered to be the central region of executive functions in humans, and patients with damage to this region may show cognitive difficulties in organizing behavioral responses, extracting memory, and generating motor programs. The meta-analysis showed that tDCS could have a significant therapeutic effect on cognitive performance. Although the sample size was relatively small, the results of this meta-analysis extend the previous findings that the tDCS protocol may improve cognitive function in PD (Doruk et al., 2014; Biundo et al., 2015). This may be related to the cortical excitability enhanced by anode tDCS (Broeder et al., 2015). Transcranial direct current stimulation, as a non-invasive method of brain stimulation, can promote cerebral cortical blood flow (Cosmo et al., 2015). Polar-dependent shifts are capable of generating resting membrane potentials at the neuronal level (Broeder et al., 2015). In relation to this, the stimulation of the cortex by tDCS may promote the neural connectivity between the cortical and subcortical network, and improve neuroplasticity in patients with PD (Bindman et al., 1964; Nitsche and Paulus, 2000). Can anode tDCS improve motor function in PD? The results of our meta-analysis showed that tDCS did not significantly improve UPDRS III, TUG, gait, or balance. We attempted to determine the reason for this from the tDCS parameters. For the effect of tDCS on the UPDRS III of PD, we performed a subgroup analysis of the stimulation duration, intensity, and session, and the same results were observed. We also compared the stimulation area of tDCS, the number of subjects included, the severity of the disease, and the status of drug treatment between the studies, and found large differences. Bueno et al. (2019) showed that a single DLPFC stimulation was insufficient to promote gait changes in PD patients, and they argued that the assessment tools used in the study do not represent the gold standard and may not be sensitive enough to detect changes. Therefore, we believe that many factors contribute to the tDCS induced improvement of motor functions in patients with PD. However, some studies have demonstrated the effectiveness of tDCS in improving the motor function of patients with PD. In Qiao and Yan (2018), the anode stimulation of the left DLPFC in terms of the step width resulted in a significant difference between tDCS and sham intervention, suggesting that tDCS intervention can improve bradykinesia in patients with early PD to a certain extent. In previous studies (Schabrun et al., 2016), the anode stimulation of tDCS was found to improve the cadence of PD. Wu and Wu (2016) and Yang and He (2017) also showed that the stimulation of the left DLPFC with anode tDCS can improve the scores of the UPDRS III in PD. In one case (Kaski et al., 2014a) report of a patient with moderate PD, while dancing the tango, the peak velocity of the trunk was significantly greater than the sham stimulation upon stimulating anode tDCS the primary and premotor cortex. This may be because tDCS intervention triggers the motor and prefrontal cortex regions, which may lead to the release of dopamine in patients with PD and promote the improvement of motor functions (Voon et al., 2009; Manenti et al., 2016; Lee et al., 2019). Another possible mechanism is that cerebellar tDCS improves sensorimotor functions through either cerebello-brain inhibition or dentate disinhibition (Ferrucci et al., 2016; Kimpel et al., 2020). Hence, there was insufficient evidence to demonstrate the effectiveness of tDCS in improving the motor functions of patients with PD. More sensitive tests, such as a three-dimensional gait analysis system or electromyography, are needed to assess motor functions in the future. This is consistent with the conclusions drawn from a review by Nardone et al. (2020). Future research should identify the optimal stimulation targets for motor function in patients with PD.

In this review, 17 studies used 2 mA stimulus intensity, and none compared the effects of different tDCS intensities on PD. Why choose 2 mA stimulation intensity? Possibly because some studies have shown that 2 mA stimulation increases cortical excitability more than 1 mA stimulation (Shekhawat et al., 2013; Murray et al., 2015). In Beretta et al. (2020b), the anode tDCS stimulation of M1 improved the ability of patients with PD to respond to external interference, and 2 mA showed more improvement than 1 mA. However, other studies have found that, for anode tDCS, regardless of the current intensity of 1 or 2 mA, the amplitudes of the motor-evoked potentials did not change significantly (Tremblay et al., 2016; Jamil et al., 2017). In addition, in our meta-analysis, the results suggested that the intensity of stimulation does not affect UPDRS III; nevertheless, high heterogeneity was noted. We conducted a subgroup analysis, which showed that a current stimulation of <2 mA had a more positive effect than the stimulation of 2 mA on the UPDRS III, which is inconsistent with the clinical observations. This may be due to the small sample size and the higher risk of bias in the included articles. Therefore, further research is needed to understand whether the intensity of tDCS is an important factor affecting the results. None of the included studies used stimulation of more than 2 mA. However, in Agboada et al. (2019), an increase in excitability was observed from the lower to higher current intensities (1 vs. 3 mA). Therefore, further studies on the tolerance and efficacy of this method should be conducted in the future.

The stimulation duration of the tDCS protocol differed among the included studies from 7 to 30 min. This large difference may explain the obvious differences in effects. Regarding the stimulation duration, although most studies used tDCS for 20 min, positive results for shorter stimulation durations have also been reported. Kaski et al. (2014b) found that placing the anode tDCS over the primary motor cortex for 15 min combined with physical activity had significant benefits for gait speed and balance. Nitsche and Paulus (2001) reported that 5–13 min of anode tDCS on the motor cortex leads to increased motor-evoked potentials. Another study reported that increasing the tDCS duration would be necessary to increase the magnitude or duration of plasticity (Tremblay et al., 2016). Agboada et al. (2019) reported that there was no significant correlation between therapeutic effect and stimulation durations. In this review, the association between stimulation duration and efficacy in patients with PD was not determined; therefore, it is necessary to conduct a more comprehensive evaluation of the effect of the duration of tDCS on PD treatment.

In most studies in this review, repeated sessions were conducted, but some studies have shown that a single tDCS on the left DLPFC in patients with PD can improve balance and functional activity (Lattari et al., 2017). A meta-analysis showed that compared with mono-target stimulations, multi-target stimulation showed significant improvements in mobility, balance, gait velocity, and fall reduction (Orrù et al., 2019). Whether single or repetitive stimulation is more effective is still uncertain, and the effect of tDCS sessions on the treatment of PD needs to be further evaluated. Compared with the therapeutic effects of tDCS on single stimulation, multiple stimulations in PD functional rehabilitation will likely become a hot research topic in the future. The PDQ-39 and UPDRS II were used to assess the quality of life of the subjects. In Manenti et al. (2018), no significant difference in the PDQ-39 scores was observed between the experimental and control groups. This may be because daily activities are complex motor manifestations that require a combination of motor and cognitive abilities, as well as other functional capabilities.

In the analysis of the included studies, several limitations affect the results. First, the quality of the studies was relatively moderate. Second, the sample size was small, making it difficult to generalize the results. Third, there was high heterogeneity in the research design of the included articles and tDCS protocol, which makes it difficult to determine the most suitable protocol for improving the clinical symptoms of PD. Finally, only Chinese and English literature were included, and the risk of article selection may affect the comprehensiveness of the results.

Therefore, in the future, more multi-level and multi-center authoritative controlled studies with large samples are needed. The determination and unification of the tDCS treatment parameters, the establishment of the sham stimulation group, and determination of the curative effect and treatment mechanism, all need to be explored in more detail, and treatment standards and norms should be issued to guide clinical practice.

## Conclusion

The results showed that tDCS appears to improve cognitive performance, but there is insufficient evidence to demonstrate that tDCS is effective in the treatment of the motor function of patients with PD. Further multicenter research with larger sample sizes is needed. Future research should focus on determining the tDCS parameters that are most beneficial to the functional recovery of patients with PD.

## Data Availability Statement

The original contributions presented in the study are included in the article/supplementary material, further inquiries can be directed to the corresponding author/s.

## Author Contributions

PW, XG, and YW: conceptualization and writing-review and editing. XL, HL, ZL, JR, JW, PW, XG, and YW: data curation. XL, HL, ZL, JR, JW, PW, and YW: formal analysis, methodology, and writing-original draft. PW and XG: investigation. HL, PW, and XG: project administration. XL, HL, ZL, and JR: software. All authors contributed to the article and approved the submitted version.

## Conflict of Interest

The authors declare that the research was conducted in the absence of any commercial or financial relationships that could be construed as a potential conflict of interest.

## Publisher's Note

All claims expressed in this article are solely those of the authors and do not necessarily represent those of their affiliated organizations, or those of the publisher, the editors and the reviewers. Any product that may be evaluated in this article, or claim that may be made by its manufacturer, is not guaranteed or endorsed by the publisher.
